# Evaluation of Treatment Decision-Making for Teeth with Post-Treatment Apical Periodontitis Among Dental Students, General Dentists, and Endodontists in Chile

**DOI:** 10.4317/jced.63471

**Published:** 2026-03-30

**Authors:** Nicolás Dufey-Portilla, Ignacia Vera, Fernando Peña-Bengoa, Giovanna Bórquez, Carolina Cáceres, Venkateshbabu Nagendrababu, Paul M. H. Dummer, Francesc Abella, Marc Garcia-Font

**Affiliations:** 1 Department of Endodontics, School of Dentistry, Universidad Andres Bello, Viña del Mar, Chile; 2 Endodontic Specialty Program, School of Dentistry, Universidad Andres Bello, Viña del Mar, Chile; 3 Private Practice, Antofagasta, Chile; 4 Department of Restorative Dentistry, College of Dental Medicine, University of Sharjah, Sharjah, UAE; 5 Department of Conservative Dentistry and Endodontics, Dr. D. Y. Patil Dental College &amp; Hospital, Dr. D. Y. Patil Vidyapeeth (Deemed to be University), Pimpri, Pune, India; 6 School of Dentistry, College of Biomedical and Life Sciences, Cardiff University, Cardiff, UK; 7 Department of Endodontics. Universitat International de Catalunya, School of Dentistry, Sant Cugat del Valles, Barcelona, Spain

## Abstract

**Background:**

This study aimed to evaluate treatment decision-making processes when assessing periapical radiographs of teeth with post-treatment apical periodontitis (AP) among dental students (DS), general dental practitioners (GDP), and endodontists (E) within Chile, and to identify demographic and tooth-related factors associated with extraction decisions.

**Material and Methods:**

A cross-sectional study was conducted between 2023 and 2024, involving 431 participants who assessed standardised periapical radiographs depicting clinical scenarios and selected one treatment option: root canal retreatment, apical surgery/intentional replantation, or extraction. A nested mixed logistic regression model and simple logistic regression analyses were performed to evaluate associations between treatment choices and clinician- or tooth-related variables (p &lt; .05).

**Results:**

Demographic factors significantly associated with extraction decisions included professional group, age, and workplace. GDP and DS were significantly more likely to recommend extraction (p &lt; 0.001 for both), particularly among GDPs aged 36-45 years (p = 0.003) and those working in the public sector. All tooth-related variables significantly influenced decisions. Teeth with indirect restorations were associated with higher extraction rates, especially among GDP (p &lt; 0.001). Inadequate root canal fillings significantly increased extraction decisions by GDPs (p &lt; 0.001). GDPs also had a strong tendency to extract teeth with medium (p &lt; 0.001) and large apical lesions (p = 0.001).

**Conclusions:**

Significant differences were observed across professional groups, with E demonstrating a greater tendency toward tooth preservation. Improving clinical decision-making in undergraduate and continuing dental education may help reduce unnecessary extractions and support evidence-based health planning in underserved areas with limited access to specialist care.

## Introduction

Post-treatment apical periodontitis (AP) is an inflammatory condition resulting from persistent microbial infection after root canal treatment (1 , 2). It is characterized by a chronic inflammatory response of the periapical tissues caused by microorganisms and their by-products originating from the root canal system (2). It can develop even when root fillings appear radiographically adequate, with a prevalence ranging from 5-15% (3). The persistence of AP in root-filled teeth has been associated with inadequate canal cleaning and shaping, incomplete canal filling, and post-treatment contamination due to coronal microleakage (4). Its management involves not only clinical and radiographic assessment but also the clinician's competence and access to appropriate treatment modalities (5 , 6). While conservative options such as retreatment, apical microsurgery, and intentional replantation are available, extraction should be considered only for cases with a poor prognosis due to its potential impact on patient quality of life (7 , 8). Tooth loss has been associated with functional decline and poorer systemic health outcomes, underscoring the importance of sound decision-making (9 , 10). However, clinical decisions in cases of AP are complex and influenced by more than evidence alone; clinician-specific variables such as professional training, clinical experience, and workplace setting also play key roles (11 - 14). Prior studies have demonstrated that endodontists (E) are more likely to select conservative treatment options, while general dental practitioners (GDP) and dental students (DS) often favor extraction (14 - 16). Clinical decision-making in cases of post-treatment AP is a multifactorial process that must consider several aspects when evaluating the therapeutic alternative. Factors related to the initial endodontic treatment, such as the quality of the root canal filling, the presence of fractured instruments, and intraradicular posts, have been shown to exert a significant influence on treatment selection (15). Likewise, the quality of the existing coronal restoration or the need for a new coronal restoration has also been identified as a relevant variable in this process (11). In this context, non-surgical retreatment is generally considered the first-line therapeutic option, with reported success rates ranging from 81-86% (15), whereas apical surgery presents success rates between 78-91% (17). In Chile, the educational landscape in endodontics has evolved in recent years, with increased adoption of new technologies and techniques (18). Nevertheless, a Chilean review reported that endodontic procedures are the leading cause of dental malpractice claims in the country, primarily due to incorrect treatment decisions and procedural errors (19). This raises concerns about whether current training adequately prepares practitioners to select conservative treatments when appropriate. Despite global evidence, national data on how treatment decisions are made across different levels of professional training remain scarce (20). Therefore, this study aimed to evaluate treatment decision-making processes when assessing teeth with post-treatment apical periodontitis among DS, GDP, and E in Chile, and to identify demographic and tooth-related factors influencing the decision to extract. The findings are intended to inform both clinical practice and dental education, particularly regarding the development of clinical reasoning and evidence-based treatment planning. The null hypothesis was that there are no significant differences in treatment decisions for post-treatment AP among DS, GDP, and E, and that these decisions are not influenced by demographic or tooth-related variables.

## Materials and Methods

**Figure 1:**
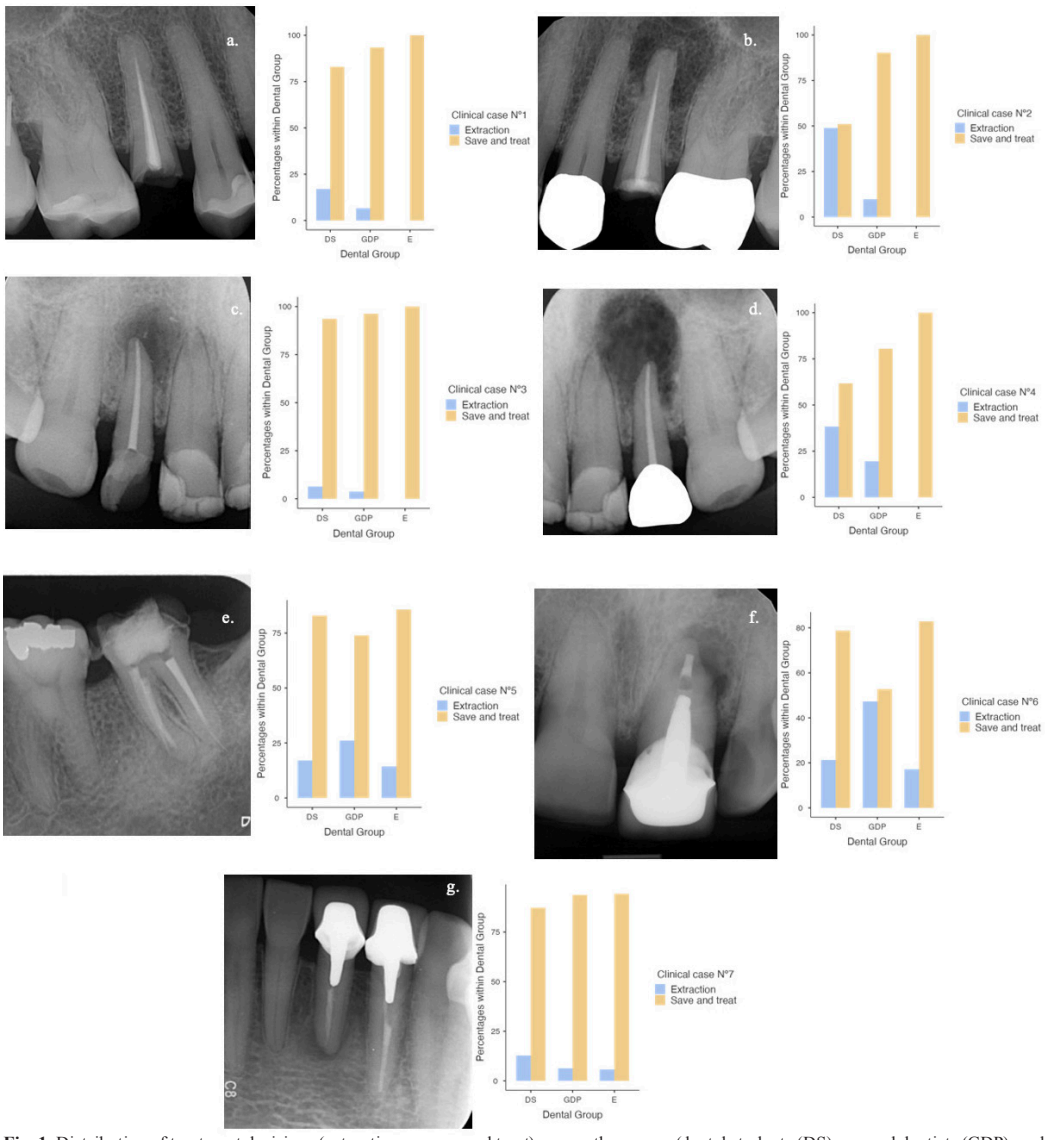
Distribution of treatment decisions (extraction vs. save and treat) among the groups (dental students (DS), general dentists (GDP), and endodontists (E)) for each of the seven clinical cases. Each case is represented by a standardised intraoral periapical radiograph and a bar graph showing the percentage distribution of treatment decisions for each group. (a) Deficient root canal filling status, no apical lesion, and no restoration. (b) Deficient root canal filling status, large apical lesion (&gt; 5 mm), and direct temporary restoration. (c) Optimum root canal filling status, large apical lesion, and direct restoration. (d) Optimum root canal filling status, large apical lesion, and indirect restoration. (e) Deficient root canal filling status, small apical lesion (&lt; 3 mm), and direct restoration. (f) Deficient root canal filling status, medium apical lesion (3-5 mm), and indirect restoration, and (g) Deficient root canal filling status, small apical lesion, and indirect restoration.

This cross-sectional, analytical, observational study was reviewed and approved by the Ethics and Scientific Committee of Universidad Andrés Bello (Chile) under Resolution 163/23. The study adhered to the Preferred Reporting Items for Observational Studies in Endodontics (PROBE) guidelines (21) and was organized by a research team affiliated with the Department of Endodontics at Universidad Andrés Bello dental school. All principal investigators were dentists with academic and clinical expertise in endodontics and epidemiological research. No clinical or laboratory interventions were performed; all data were collected remotely through anonymous surveys distributed nationwide. - Study participants The sample size was calculated using a 95% confidence level, 5% significance level, and 5% precision, based on the total number of DS, GDP, and E in Chile. Population estimates were obtained from the Chilean Superintendence of Health and the College of Dental Surgeons (22 , 23). The estimated population included 1,600 final-year DS (national average of annual graduates), 28,495 GDP, and 1,848 E, yielding a total of 31,943 individuals. This calculation resulted in a minimum sample size of 379 participants, proportionally distributed across the three groups. However, a positive response rate of 444 participants (99.7%) allowed the final sample to be increased to 431 participants (47 DS, 349 GDP, and 35 E). Thirteen responses were excluded due to non-compliance with inclusion criteria and/or incomplete information. It is important to note that the study did not assess 431 real cases of post-treatment AP, but rather 431 clinician responses to hypothetical scenarios. Inclusion criteria were: (1) final-year dental students (DS); (2) general dental practitioners (GDP) registered with the Superintendence of Health and having completed a minimum of six years of undergraduate dental education; and (3) endodontists (E) with at least two years of formal postgraduate training in endodontics. Exclusion criteria included: DS below their final year of training, GDP currently enrolled in postgraduate endodontic programs, and GDP registered in short-term endodontic training courses. - Data collection A survey was conducted anonymously between July and September 2023. Digital or written informed consent was obtained from all participants before their inclusion in the study. The survey was distributed digitally via Google Forms and in person at various academic institutions and private and public health centers. Each format included a presentation letter, a description of the study, and an informed consent document. Participants accessed the digital version through a QR code or email link. Numbered forms were provided for face-to-face interviews. Data were recorded in Google Sheets. Although the survey was coordinated by a single academic institution, it was distributed nationally, and participants were recruited from multiple regions, dental schools, and clinical settings across Chile. - Survey Instrument The survey anonymously assessed treatment decisions for post-treatment AP using seven standardised periapical radiographs manipulated with Adobe Photoshop software (Fig. 1).

1 Screenshot

The questionnaire comprised two sections and 11 items in total: (1) clinician characteristics (professional group, age category, years of experience, and workplace setting) and (2) seven case-based items consisting of standardised periapical radiographs, each followed by a single forced-choice question requiring selection of one treatment option (A-C). The survey was developed using a variable-controlled case-scenario approach commonly applied in endodontic decision-making research (6). Content relevance and face validity were ensured through iterative review within the author team (including endodontists and epidemiology/statistics expertise) and refinement of wording and layout to optimize clarity and completion. Because the instrument was designed to elicit discrete case-based treatment decisions rather than measure a latent psychometric construct, internal consistency testing was not applicable; the primary focus was standardization of scenarios and response options. Each case presented the same clinical scenario: a 50-year-old patient with no systemic diseases experiencing pain on biting associated with a previously root-filled tooth with a positive percussion test and normal adjacent soft tissues. Participants were asked to select one of the following three treatment options: Option A: Root canal retreatment; Option B: Apical surgery/intentional replantation; Option C: Extraction. Participants were asked to choose only one option among root canal retreatment, apical surgery/intentional replantation, or extraction. It was assumed that respondents selecting a conservative approach might perform the treatment themselves or refer the case, depending on their clinical scope. In all scenarios, post-treatment AP was operationally defined as a symptomatic, previously root-filled tooth presenting a periapical radiolucency on periapical radiographs. No information was provided regarding the time elapsed since the original treatment because this factor can introduce heterogeneity and anchoring bias in case-based surveys, given that follow-up intervals and healing expectations vary across clinicians and healthcare settings. While radiographic healing after root canal treatment is time-dependent and may continue over extended periods, including an elapsed-time variable could have confounded group comparisons by shifting respondents' interpretation toward disparate follow-up protocols rather than the predefined radiographic and restorative variables under evaluation. This design choice was therefore used to standardize the scenarios and isolate decision patterns based on radiographic interpretation and professional reasoning, and it is acknowledged as a limitation in the Discussion. Each case incorporated additional variables reflecting endodontic and restorative status. Root canal filling quality was assessed according to the European Society of Endodontology guidelines (24). The type of restoration (direct or indirect), presence or absence of a periapical lesion, and lesion size (small &lt; 3 mm, medium 4-5 mm, large &gt; 5 mm) were also considered. Although these lesion size ranges were applied during study design and analysis, they were not explicitly provided to participants. Instead, the radiographs were intended to visually communicate these differences. Participant demographic information (age, years of experience, and workplace: public vs private) was also collected to examine potential influences on decision-making. - Data analysis The analysis followed a multi-step approach. First, responses were classified as tooth-preserving ('save and treat': options A or B) or 'extract' (option C). Because each participant evaluated seven cases (repeated measures), associations with extraction were primarily assessed using a generalized linear mixed-effects logistic regression model (logit link; binomial distribution) with a random intercept for participant to account for within-respondent correlation. Fixed effects included professional group (DS, GDP, and E) and clinician- and tooth-related covariates (age category, years of experience, workplace setting, lesion size category, restoration type, and radiographic root canal filling status). Comparisons between professional groups and covariate levels were based on Wald tests of the regression coefficients, and results are reported as odds ratios (OR) with 95% confidence intervals (CI). In addition, simple logistic regression analyses were used to explore group-specific associations and facilitate the interpretation of extraction tendencies for each variable. Two-sided p-values &lt; 0.05 were considered statistically significant. All analyses and visualizations were conducted using R statistical software version 4.3.1 (2023-06-16 ucrt).

## Results

The survey achieved a 99.7% response rate, with participants from all 16 regions of Chile. Table 1 summarizes the demographic characteristics of the participants across groups.

Table 1

**Figure 2:**
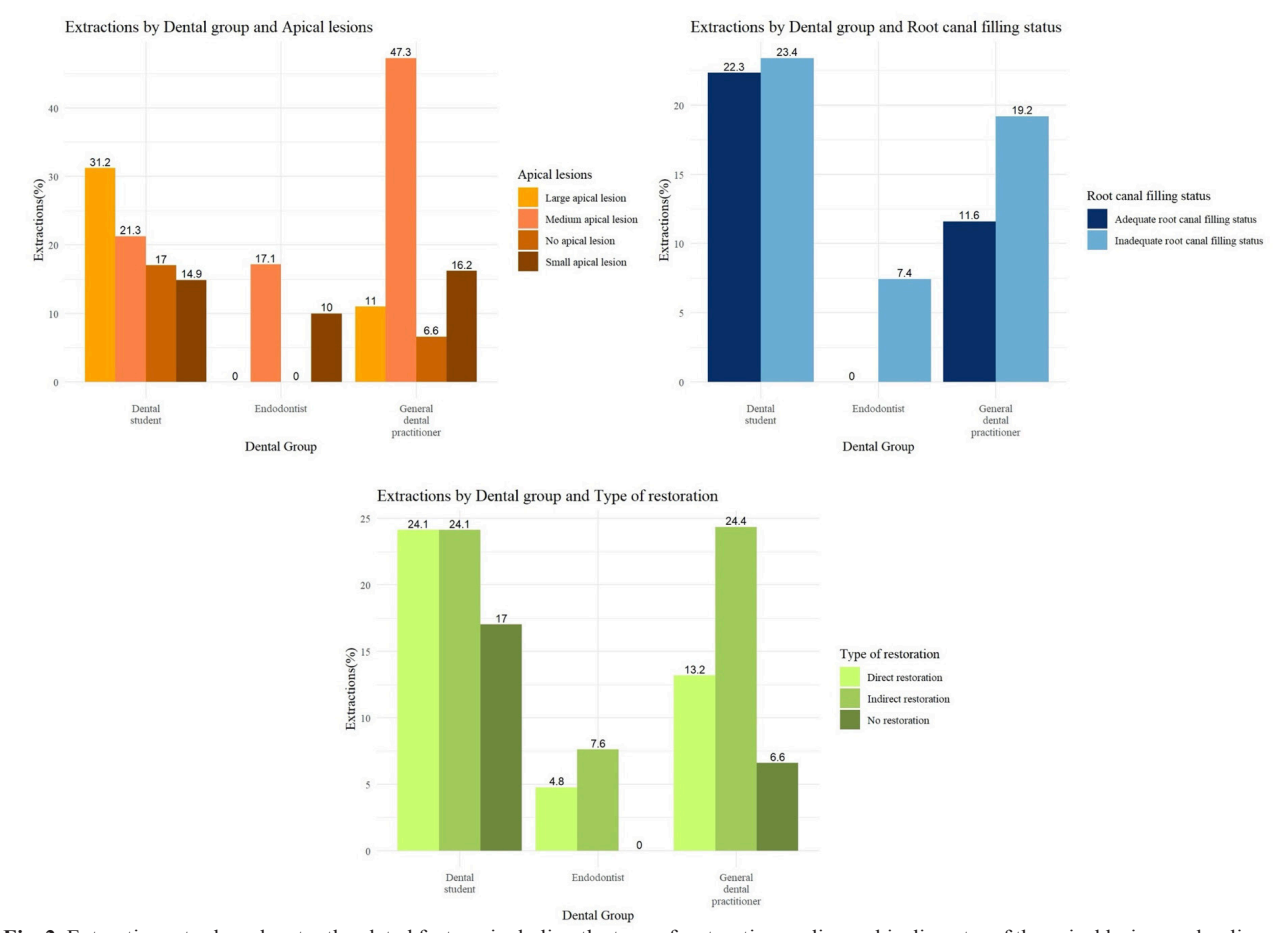
Extraction rates based on tooth-related factors, including the type of restoration, radiographic diameter of the apical lesion, and radiographic status of the root canal filling.

Figure 1 shows the distribution of all three treatment options (root canal retreatment, apical surgery/intentional replantation, and extraction) across the seven scenarios and professional groups. In line with the study objective of identifying factors associated with the decision to extract, inferential analyses focused on extraction (option C) versus tooth-preserving decisions ('save and treat'; options A or B). When responses were dichotomized into tooth-preserving ('save and treat') versus extraction, demographic factors significantly associated with extraction decisions included the dental group, age (36-45 years), and workplace setting. Both GDP (OR = 4.37, p &lt; 0.001) and DS (OR = 4.41, p &lt; 0.001) had a significantly higher likelihood of performing extractions compared to E. GDP aged 36-45 years were also significantly more likely to extract (OR = 1.99, p = 0.003). Regarding workplace setting, GDP working in the public sector were more likely to perform extractions (22.96%) compared to those in private clinics (15.24%) or mixed environments (15.95%). However, neither DS nor E were represented in the public sector, limiting comparative analysis across all professional groups. Tooth-related variables also significantly influenced extraction decisions, particularly the type of restoration, radiographic size of the apical lesion, and radiographic status of the root canal filling. Figure 2 displays the extraction percentages by group and variable. Extraction rates were highest for teeth with indirect restorations, especially among GDP (24.35%, OR = 2.12, p &lt; 0.001) and DS (24.11%). Teeth with inadequate radiographic root canal filling status were associated with significantly higher extraction rates among GDP (OR = 1.81, p &lt; 0.001), whereas no significant differences were observed among DS or E. Regarding the radiographic size of apical lesions, GDP had a marked tendency to extract in the presence of medium-sized lesions (OR = 7.26, p &lt; 0.001) and large lesions (OR = 1.56, p = 0.001). DS also demonstrated a preference for extraction in cases with large lesions. In contrast, no E opted for extraction regardless of lesion size, reflecting a more conservative treatment approach. Complete statistical results are presented in Table 2 and (Supplement 1) http://www.medicinaoral.com/medoralfree01/aop/jced_63471_s01.

Table 2

The results, presented in (Supplement 1) http://www.medicinaoral.com/medoralfree01/aop/jced_63471_s01, are visually summarized in Figure 2, which provides a concise overview of these trends, facilitating accessibility and comparison across variables and professional groups.

2 Screenshot

## Discussion

The present study investigated the treatment decisions for post-treatment apical periodontitis (AP) among final-year dental students (DS), general dental practitioners (GDP), and endodontists (E) in Chile. Management of post-treatment AP is challenging, as the available evidence does not clearly support the superiority of surgical over non-surgical approaches for optimal outcomes, as highlighted in a Cochrane review (25). This underscores the importance of individualized and evidence-based clinical decision-making that considers patient-specific and contextual variables. Apical periodontitis associated with root-filled teeth is a common finding in epidemiological studies (2), making it a frequent clinical outcome and a potential driver of tooth loss. Moreover, AP has been associated with systemic inflammatory burden, reinforcing the importance of accurate diagnosis and appropriate treatment planning (26). These differences are clinically relevant in settings where access to specialist endodontic care may be constrained, because early decision thresholds can disproportionately shape tooth-preservation versus extraction pathways. The study used a nationwide sample and seven standardised clinical scenarios, applying a structured, variable-controlled vignette design to isolate how prespecified radiographic and restorative features influence management choices for post-treatment apical periodontitis and to minimize information bias by improving comparability across respondents (6 , 10 , 11 , 14 , 15). Clinical information was intentionally standardised and restricted to reduce uncontrolled assumptions (e.g., symptoms, restorability, patient preferences, costs, and access/referral constraints), allowing differences to be more plausibly attributed to radiographic/restorative cues and the training background of the clinicians; this design may increase reliance on radiographic appearance, which mirrors primary-care conditions where decisions often start from periapical radiographs and limited chairside data. The response options reflected a pragmatic tooth-preservation hierarchy: non-surgical retreatment as first-line when restorable and intra-radicular infection is suspected; apical surgery/intentional replantation when retreatment is unlikely or unfeasible; and extraction as a last-line option for non-restorable teeth, severe structural compromise, or unfavorable prognosis (17 , 24 , 25 , 27 , 28). Time since the original treatment was omitted to avoid heterogeneous respondent assumptions, although it remains clinically relevant and should be incorporated in future research. The findings align with international trends and highlight significant decision-making variations influenced by professional experience, age, workplace, and clinical case complexity. For instance, Rodríguez et al. (29) investigated the impact of cone beam computed tomography (CBCT) on the clinical decision-making of GDP and E following failed root canal treatment. In their study, E and GDP first analyzed eight clinical cases with a range of variables; similar to the present study, using clinical and radiographic information, 5.63% of E and 14.69% of GDP opted for tooth extraction, with GDP having a threefold higher extraction rate. This corresponds with the present findings, where E demonstrated a significantly greater tendency to preserve teeth, with extraction rates as low as 5.3%. This aligns with their advanced training and familiarity with alternative procedures, such as apical microsurgery and intentional replantation, which are associated with high success rates (78.4-91.3% and 88-98%, respectively) (17 , 27). In contrast, GDP and DS opted for extraction more frequently, with GDP recommending higher extraction rates (17.03%). This discrepancy may reflect a lack of awareness or confidence in conservative treatment options (26). Tooth-related factors played a significant role in treatment decision-making across all groups. For instance, teeth with direct restorations were less likely to be extracted compared to those with indirect restorations, where GDP exhibited the highest extraction rate (24.35%). This preference might reflect the perceived restorative complexity and the limited exposure of GDP to interdisciplinary planning approaches more common among specialists. Deficient root canal fillings significantly influenced extraction decisions, particularly among DS, whose extraction rate reached 23.4%. E were less affected by this variable (7.43%), possibly due to their expertise in retreatment techniques (26). These results are consistent with those reported by Lee et al. (6), where teeth with underfilled root canals had higher extraction rates among GDP. Similarly, the size of the periapical lesion emerged as a key variable influencing treatment decisions, particularly among DS and GDP. These groups had a marked tendency to extract teeth with medium or large lesions, likely associating larger radiolucencies with a poorer prognosis. Although lesion size is not, in itself, a reliable indicator for retreatment or extraction (29), its inclusion in this study was intended to evaluate how radiographic features shape clinical perception, especially among less-experienced clinicians. These findings underscore the need to enhance undergraduate and continuing dental education by strengthening radiographic interpretation skills and promoting evidence-based criteria for treatment planning, with the ultimate goal of improving tooth preservation rates. In addition to tooth-related variables, the present study observed varying percentages of tooth-saving and extraction rates depending on age, years of experience, and workplace. Age and professional group played significant roles. Regardless of the partial tendency for younger professionals to perform fewer extractions, GDP aged 36-45 years were more likely to extract teeth (p = 0.003). This finding contrasts with studies from other countries, where more experienced GDP tended to extract more frequently (2). This may be related to the different public health policies in other countries, private systems, and university programs. Research and analysis of each region of the world are essential to follow and replicate those with regions where tooth retention is preferred to provide patients with the best possible options while recognizing the long-term benefits of tooth preservation for the stomatognathic system and overall patient well-being. In this context, the inclusion of final-year DS aimed to capture decision-making tendencies at the threshold between academic learning and clinical practice. While their limited experience may result in a narrower interpretation of diagnostic scenarios, it provides valuable insight into how clinical reasoning evolves with exposure. This underscores the importance of strengthening integrative treatment planning before graduation. In the present study, no statistically significant differences were observed between years of professional experience and dental groups. This contrasts with findings from studies such as Rodríguez et al. (26), which reported that clinical experience significantly influences decision-making in cases of AP in other countries. Nevertheless, descriptive data revealed a tendency among GDP with 20 or more years of experience to favor tooth preservation more frequently than their less experienced peers. While this trend did not reach statistical significance, it may reflect greater clinical confidence, broader procedural knowledge, and a long-term perspective. The workplace also influenced decisions, with GDP in the private sector having a greater tendency to preserve teeth (15.24% extraction rate) compared to those in the public system (22.96% extraction rate). This difference can be attributed to several factors related to the characteristics of the two Chilean systems. As reported by Lixandru et al. (30), the public dental system involves long waiting times for consultation and specialist treatment, which might impact decision-making by GDP within the public system. These long waiting times may exacerbate symptoms or disease progression, leading patients to seek immediate solutions. As a result, tooth extraction might often be preferred. From a health-systems perspective, the higher extraction propensity among GDP and DS is relevant in settings where access to specialist endodontic care and advanced modalities is constrained. Under such constraints, treatment decisions may rely on lower thresholds for extraction and radiographic heuristics, potentially increasing the risk of avoidable tooth loss and subsequent restorative needs. Given the scarcity of regional evidence, these findings provide baseline data to inform locally adapted curricula, continuing professional development, and referral pathways. This study has several limitations. First, only tooth-related variables were analyzed, excluding patient-related factors such as systemic health, lifestyle, and socioeconomic status, which can influence treatment outcomes and clinical decisions (26). Future studies should incorporate these elements to provide a more comprehensive understanding of decision-making. Second, CBCT, a more accurate diagnostic tool for periapical pathosis, was not used, reflecting its limited availability in Chile's public dental system. While this decision aimed to simulate common local conditions, it may limit the generalizability of the findings to contexts where advanced imaging is routinely available. Third, the approximate time elapsed since the original root canal treatment was not specified in any of the scenarios. While this omission was deliberate to ensure uniformity across cases and limit extraneous variability, it may have affected the respondents' ability to fully assess prognosis, especially among more experienced clinicians, who typically consider treatment age when evaluating long-term outcomes. Future studies should incorporate this variable to better align with the standard of care and enhance the clinical realism of hypothetical decision-making. Lastly, comparisons with other South American countries were not feasible due to the limited availability of comparable data. Future studies should consider multicentric approaches that explore regional trends and inform context-specific educational and clinical strategies. The present study highlights the need to harmonize decision-making between the three dental groups. While GDP may be aware of the best treatment options, systemic and contextual limitations can hinder their ability to implement them, particularly in the public sector. Despite their theoretical knowledge, these constraints may affect their confidence and competence in managing complex cases. Ultimately, the study underscores the complexity of clinical decision-making in managing post-treatment AP and stresses the necessity of ongoing professional development. It also highlights the importance of providing GDP and DS with the necessary tools, training, and support to execute evidence-based practices effectively, ensuring optimal patient outcomes across all dental care sectors. These findings have direct implications for dental education. The significant variation in treatment decisions among DS and general practitioners highlights the need to strengthen clinical reasoning and endodontic diagnostic skills during undergraduate and continuing education. Integrating decision-making frameworks, guided case-based learning, and evidence-based protocols into the curriculum may help reduce variability, improve treatment outcomes, and foster a greater commitment to tooth preservation. Presenting final-year DS with standardised diagnostic scenarios, especially when followed by specialist-led feedback, may further enhance their clinical judgment and promote reflective learning. Incorporating such strategies into formal curricula may also strengthen the transition from academic training to independent clinical practice. Such interventions are essential to foster competent, confident clinicians equipped to manage complex endodontic cases and avoid unnecessary extractions.

## Conclusions

Treatment decisions for radiographic evidence of post-treatment apical periodontitis differed across professional groups in Chile. Compared with endodontists, general dental practitioners and final-year dental students were significantly more likely to recommend extraction; among general practitioners, clinician age and workplace setting were also associated with extraction. Scenario characteristics related to restoration status, root filling appearance, and periapical lesion characteristics further shaped extraction choices, suggesting greater reliance on radiographic cues among non-specialists. These findings provide baseline regional evidence and support targeted educational strategies to strengthen evidence-based case assessment and referral, potentially reducing avoidable extractions where specialist access is limited.

## Figures and Tables

**Table 1:** Characteristics of the sample (variables), including the count and percentage of participants for each group.

	Variables	Number of participants % (N)
Dental group	Age	-
Dental student	23-35	100% (47)
	36-45	0
	46-55	0
	56 or more	0
GDP	23-35	63.04% (220)
	36-45	26.07% (91)
	46-55	7.45% (26)
	56 or more	3.44% (12)
Endodontist	23-35	51.43% (18)
	36-45	37.14% (13)
	46-55	11.43% (4)
	56 or more	0
Dental group	Years of experience	-
Dental student	Dental student	100% (47)
	0-5	0
	6-19.	0
	20 or more	0
Endodontist	Dental student	0
	0-5	40% (14)
	6-19	11.43% (4)
	20 or more	48.57% (17)
GDP	Dental student	0
	0-5	51.57% (180)
	6-19	12.32% (43)
	20 or more	36.10% (126)
Dental group	Workplace	-
Dental student	Dental student	100% (47)
	Mixed workplace	0
	Private sector	0
	Public sector	0
Endodontist	Dental student	0
	Mixed workplace	45.71% (16)
	Private sector	54.28% (19)
	Public sector	0
GDP	Dental student	0
	Mixed workplace	20.06% (70)
	Private sector	71.91% (251)
	Public sector	8.02% (28)

1

**Table 2:** Results from Nested Mixed Logistic Regression Analysis.

Variable	%(n)	Adjusted OR (95% CI)	p Value
Intercept	-	0.18 (0.115–0.272)	2.56E-15*
Dental Group			
Endodontist (Reference)	5.31% (13)	-	-
Dental Student	23.1% (76)	4.41 (1.83–10.65)	9.39E-04*
General Dental Practitioner	17.03% (416)	4.37 (2.25–8.48)	1.27E-05*
Age			
23–35 (Reference)	16.14% (322)	-	-
36–45	18.13% (132)	1.99 (1.25–3.17)	3.57E-03*
46–55	16.19% (34)	1.83 (0.76–4.42)	1.76E-01
56 or more	20.24% (17)	2.19 (0.73–6.57)	1.59E-01
Years of Experience			
0–5 (Reference)	15.91% (216)	-	-
6–19	15.48% (155)	0.67 (0.431–1.03)	7.20E-02
20 or more	17.63% (58)	0.62 (0.263–1.45)	2.68E-01
Workplace			
Public Sector (Reference)	22.96% (45)	-	-
Mixed Workplace	15.95% (96)	0.68 (0.387–1.19)	1.73E-01
Private Sector	15.24% (288)	0.59 (0.36–0.96)	3.44E-02*
Apical Lesions			
Small Apical Lesion (Reference)	15.55% (134)	-	-
Medium Apical Lesion	42.0% (181)	4.70 (3.4–6.5)	6.93E-21*
No Apical Lesion	7.19% (31)	0.37 (0.24–0.59)	1.80E-05*
Large Apical Lesion	12.30% (159)	0.77 (0.53–1.13)	1.87E-01
Type of Restoration			
Direct Restoration (Reference)	13.69% (177)	-	-
Indirect Restoration	22.97% (297)	0.96 (0.72–1.27)	7.70E-01
Radiographic Status of Root Canal Filling			
Adequate Root Canal Filling Status (Reference)	11.83% (102)	-	-
Inadequate Root Canal Filling Status	18.70% (403)	1.22 (0.76–1.65)	5.57E-01

Notes:• CI: Confidence Interval.• Adjusted OR: Odds Ratio adjusted using nested mixed logistic regression analysis.• P Value: Statistical significance (* indicates p < 0.05)

## Data Availability

The datasets used and/or analyzed during the current study are available from the corresponding author.
